# Efficacy of sulphadoxine-pyrimethamine for intermittent preventive treatment of malaria in pregnancy, Mansa, Zambia

**DOI:** 10.1186/1475-2875-13-227

**Published:** 2014-06-09

**Authors:** Kathrine R Tan, Bonnie L Katalenich, Kimberly E Mace, Michael Nambozi, Steve M Taylor, Steven R Meshnick, Ryan E Wiegand, Victor Chalwe, Scott J Filler, Mulakwa Kamuliwo, Allen S Craig

**Affiliations:** 1 Malaria Branch, US Centers for Disease Control and Prevention, Atlanta, GA, USA; 2 Tulane University School of Medicine, New Orleans, LA, USA; 3 Tropical Diseases Research Center, Ndola, Zambia; 4 Duke University Medical Center, Durham, NC, USA; 5 University of North Carolina, Gillings School of Global Public Health, Chapel Hill, NC, USA; 6 Maina Soko Military Hospital in Lusaka, Lusaka, Zambia; 7 The Global Fund to Fight AIDS, TB, and Malaria, Geneva, Switzerland; 8 National Malaria Control Centre, Lusaka, Zambia; 9 Vaccine Preventable Disease Eradication and Elimination Branch, US Centers for Disease Control and Prevention, Atlanta, GA, USA

**Keywords:** Malaria, Pregnancy, Intermittent-preventive treatment, Sulphadoxine-pyrimethamine

## Abstract

**Background:**

Intermittent preventive treatment of malaria in pregnancy (IPTp) with sulphadoxine-pyrimethamine (SP) decreases adverse effects of malaria during pregnancy. Zambia implemented its IPTp-SP programme in 2003. Emergence of SP-resistant *Plasmodium falciparum* threatens this strategy. The quintuple mutant haplotype (substitutions in N51I, C59R, S108N in *dhfr* and A437G and K540E in *dhps* genes), is associated with SP treatment failure in non-pregnant patients with malaria. This study examined efficacy of IPTp-SP and presence of the quintuple mutant among pregnant women in Mansa, Zambia.

**Methods:**

In Mansa, an area with high malaria transmission, HIV-negative pregnant women presenting to two antenatal clinics for the 1^st^ dose of IPTp-SP with asymptomatic parasitaemia were enrolled and microscopy for parasitaemia was done weekly for five weeks. Outcomes were parasitological failure and adequate parasitological response (no parasitaemia during follow-up). Polymerase chain reaction assays were employed to distinguish recrudescence from reinfection, and identify molecular markers of SP resistance. Survival analysis included those who had reinfection and incomplete follow-up (missed at least one follow-up).

**Results:**

Of the 109 women included in the study, 58 (53%) completed all follow-up, 34 (31%) had incomplete follow-up, and 17 (16%) were lost to follow-up after day 0. Of those who had complete follow-up, 15 (26%, 95% confidence interval [CI] [16–38]) had parasitological failure. For the 92 women included in the survival analysis, median age was 20 years (interquartile range [IQR] 18–22), median gestational age was 22 weeks (IQR range 20–24), and 57% were primigravid. There was no difference in time to failure in primigravid versus multigravid women. Of the 84 women with complete haplotype data for the aforementioned loci of the *dhfr* and *dhps* genes, 53 (63%, 95% CI [50–70]) had quintuple mutants (two with an additional mutation in A581G of *dhps*). Among women with complete follow-up and quintuple mutants, 22% had parasitological failure versus 0% without (p = 0.44).

**Conclusions:**

While underpowered, this study found 26% failure rates of SP given the moderate prevalence of the quintuple mutant haplotype. Despite the presence of resistance, SP retained some efficacy in clearing parasites in pregnant women, and may remain a viable option for IPTp in Zambia.

## Background

In sub-Saharan Africa, there are approximately 30 million pregnant women at risk for malaria [1], and the prevalence of malaria in pregnancy is estimated to be about 28% [2]. Malaria infection in pregnancy is associated with severe maternal anaemia, placental parasitaemia, low birth weight (LBW), and increased perinatal mortality [2]. To reduce the risk of poor outcomes, the World Health Organization (WHO) recommends intermittent preventive treatment of malaria in pregnancy (IPTp), specifically with sulphadoxine-pyrimethamine (SP). Sulphadoxine-pyrimethamine for IPTp (IPTp-SP) is given without determining parasitaemia status as it will either treat patients with parasites or provide a prophylactic effect to non-infected patients. At the time of the study, WHO recommended at least two doses of SP spaced one month apart starting in the second trimester [3].

In Zambia, it is estimated that there are more than 200,000 pregnancies at risk for malaria each year [4]. National guidelines for IPTp-SP at the time of the study had been implemented since 2004 and called for three doses of SP spaced one month apart, given after quickening [5]. Coverage of IPTp-SP in Zambia is among the highest in sub-Saharan Africa with 89% of pregnant Zambian women receiving any IPTp-SP and 70% of women receiving at least two doses [6,7].

The efficacy of the IPTp-SP strategy is threatened by increasing SP resistance. In Zambia, SP was first used in the late 1990s as a second-line drug for chloroquine failures. With increasing SP resistance, in 2002 artemether-lumefantrine became the first-line drug and SP was used to treat malaria only in children <5 kg and pregnant women, and for IPTp [8]. *In vivo* SP efficacy studies done in Zambia among children <5 years old demonstrated an increase in SP treatment failures from 3% (1996) to 23% (2007) in Chipata, and from 14.5% (2003) to 47% (2007) in Mansa [9-11].

It is unknown what level of parasite resistance to SP would render the drug ineffective for IPTp. Sulphadoxine-pyrimethamine resistance levels were previously monitored via *in vivo* clinical efficacy studies in children aged 6–59 months because they have not yet fully acquired immunity to malaria. Translating *in vivo* results among sick children to SP efficacy in asymptomatic pregnant women, who likely have some immunity to malaria, is challenging. Furthermore, while *in vivo* efficacy of SP in children is defined in terms of treatment efficacy, efficacy of SP-IPTp is demonstrated by clearing of initial parasitaemia, and prevention of recurrent infections because SP-IPTp’s action is both therapeutic and prophylactic. A meta-analysis of clinical studies demonstrated that IPTp-SP efficacy is maintained even in areas with as high as 39% SP treatment failure in young children [12]. *In vivo* IPTp-SP efficacy studies in pregnant Zambian women are lacking. There is one published study, a randomized controlled trial of the efficacy of 2 dose versus monthly IPTp-SP, but this study focused on HIV-positive women and was done in 2003–2004 before resistance was firmly entrenched [13].

The presence of *P. falciparum* dihydropteroate synthase (*dhps*) and dihydrofolate reductase (*dhfr*) mutations are associated with SP drug resistance in the parasite. The quintuple mutant haplotype, consisting of the N51I, C59R, S108N substitutions in *dhfr* and the A437G and K540E substitutions in *dhps* is a marker for SP treatment failure in non-pregnant patients [14]. Furthermore, presence of the I164L substitution in *dhfr* or the A581G substitution in *dhps* is associated with reduced efficacy of SP *in vitro* and *in vivo*[15-17]. There are few published studies looking at molecular markers for SP resistance in Zambia. One study of the general community in Macha, Zambia in 2006 found the quintuple marker in 6.5% of samples, and no I164L mutations, compared to the absence of these markers in 2000 in the same community [18]. There have been no published studies of the presence of these markers in parasitaemic pregnant Zambian women.

Determining the threshold level of SP resistance, in terms of prevalence of molecular markers that would render the IPTp-SP strategy ineffective could be used to predict outcomes in pregnant women receiving IPTp-SP. Furthermore, IPTp-SP efficacy in HIV-negative pregnant women in Zambia and the corresponding presence of SP resistance markers in malaria parasites infecting these women are unknown. The objectives of this study were to determine the efficacy of IPTp-SP to clear peripheral parasites in parasitaemic pregnant women, and assess the presence of SP-resistance genotypes among pregnant women in Mansa, Zambia. Mansa is in Luapula Province, the province with the highest prevalence of parasitaemia in children under five years of age in the country at 21.8% in 2008, around the time of the study [6].

## Methods

The target population was pregnant women attending antenatal clinics (ANC) between January 2010 and May 2011 at two health facilities in Mansa, Zambia. Women who were HIV negative, presented after quickening, and had received no prior anti-malarials including IPTp-SP during the current pregnancy were eligible for enrollment. Written informed consent was obtained from all participants.

Upon presentation, eligible women were asked about a history of fever, asked if they took anti-malarials within the month before enrollment, had a physical examination, which included axillary temperature, weight and fundal height, and were screened with a rapid diagnostic test (RDT) (Clearview® Malaria Combo, Orgenics Ltd, Alere Diagnostics, Yavne, Israel) that detects histidine rich protein 2 for *P. falciparum* and the pan-*Plasmodium* antigen lactate dehydrogenase. If asymptomatic parasitaemia was found on RDT, the patient was enrolled, a malaria smear was done to confirm RDT findings, and blood was collected on filter paper (FTA® Elute Cards, Whatman, Maidstone, United Kingdom) for PCR of molecular markers. Fingerprick blood samples were used to make thick and thin smears, slides were stained with a 5% Giemsa solution, and then trained laboratory technicians read thick smears for the presence of *Plasmodium* parasites, and thin smears for the quantification of intracellular parasites per microlitre (assuming total white cell count of 8,000/microlitre) [19]. All slides were re-read for quality control at a national reference laboratory. Due to the study site being remotely located from the reference lab, results of confirmatory blood smear were not available on day 0. If the day 0 smear was negative, or later found to be negative during quality control reads of the slides, the patient was excluded from the study, so that the final sample includes only women with peripheral parasitaemia detectable by blood slide microscopy on day 0.

Women received three SP tablets (500 mg sulphadoxine and 25 mg pyrimethamine per tablet), under direct observation on day 0. To ensure drug quality, all SP used in the study was procured from Roche Pharmaceuticals via a supplier from the UK. Patients were instructed to return to ANC on a weekly basis for a total of five weeks. To encourage follow-up, women were given the equivalent of $7 (USD) at the follow-up visits to cover travel expenses, and a community health worker visited the homes of women who missed their follow-up appointment to remind them about their appointments. Each follow-up visit included an interview for fever and recent medication history including anti-malarials, measurement of axillary temperature, and collection of blood for blood smears, haemoglobin measurement, and filter paper for polymerase chain reaction (PCR) to be done if the blood smear was positive. Women found to be parasitaemic on follow-up were given artemether-lumefantrine according to national guidelines. For quality control, all blood smears were read by two laboratory technicians, and all smears were reviewed by a senior laboratory technician at the national level who determined the final result. The primary endpoint was development of parasitaemia during the follow up period.

To distinguish between reinfection versus recrudescence among those with parasitaemia during follow-up, PCR with gel electrophoresis was performed on parasites from day 0 and the day of failure to compare the genetic markers for merozoite surface protein 1 (*msp1*), merozoite surface protein 2 (*msp2*), and glutamate-rich protein (*glurp*). The different allelic types identified for *msp1* (K1, MAD 20, and RO33 types) and *msp2* (FC27 and IC/3D7) were detected with specific primers in a second nested PCR. More details of the methods used have been described elsewhere [20].

Reinfection was defined as having completely different alleles between parasites from day 0 and day of failure. If any similar allele was found between the day 0 and day of failure parasite, this was considered recrudescence.

Main outcomes were classified as follows. Presence of any parasitaemia during follow-up was categorized as “parasitological failure”. Parasitological failures were further described as reinfections or recrudescences as distinguished by PCR as described above. The presence of no parasitaemia during follow-up was called “adequate parasitological response”. Women who were enrolled at day 0, had at least one follow-up day, but were not present for subsequent follow-up visits were classified as “incomplete follow-up.” Women who did not have at least one follow-up visit after day 0 were called “lost to follow-up”.

Sample size and power was calculated as follows. The expected proportion of parasitic failure in pregnant women is unknown because of limited data. An *in vivo* study in Zambian children (age <5) in 2006 showed SP resistance, defined as treatment failure excluding reinfection (PCR adjusted), to be 33% [21]. Another study, a comprehensive literature review, examined the proportional reduction in parasitaemia in women at delivery compared with SP resistance reported in symptomatic children with day 14 treatment failure, and found that the reduction was more than 60% in resistances ranging between 3–39% [12]. Since treatment failure in children <5 years old does not correlate well with treatment failure in pregnant women, and based on the findings of the literature review, it was estimated that the proportion of parasitological failures would be much lower, around 10%. To make an estimate of parasitic failure within 5% of the true value (precision = 0.05) with 95% confidence, and to achieve a power of 0.80, 138 patients was the target sample size.

Characteristics of patients and their outcomes were described using medians and frequencies. Survival analysis was done to look at time to development of parasitaemia among those who had parasitological failure or incomplete follow-up, and the log-rank test was used to test for differences between primigravid and multigravid women [22]. This analysis was also done for the PCR-adjusted outcomes in which recrudescences were considered failures, while reinfections were censored. Analyses were performed in SAS software version 9.3 and all tests were performed at the 5% level of significance [23]. The ggplot2 package in R version 3.0.1 (R foundation for statistical computing, Vienna, Austria) was used to create the Kaplan Meier figure [24].

All available day 0 specimens for women included in the study underwent PCR to detect mutations on the genes for *dhps* and *dhfr* using amplification, Sanger sequencing, and manual scoring of chromatographs at the loci of interest, as described elsewhere [25]. Specifically, mutations on the S108N, N51I, C59R, and I164L positions of the *dhfr* gene, and A437G, K540E, and A581G positions of the *dhps* gene were targeted. Mixed alleles were classified as mutant. The presence of the double, triple, and quintuple mutants was examined. The proportion of parasitological failure in women with these mutations versus without mutations was compared with Pearson chi-squared tests.

Ethical approval for this study was obtained from institutional review boards at both the US Centers for Disease Control and Prevention and the Tropical Diseases Research Centre in Zambia.

## Results

**Figure 1:**
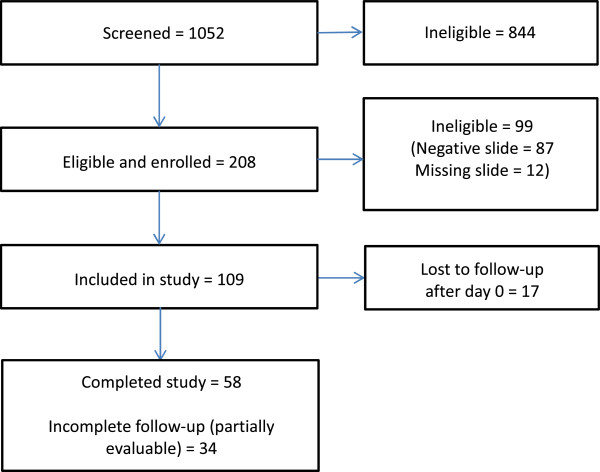
Flow diagram of enrollment and inclusion of participants.

Of the 1,052 women screened, 208 had positive RDTs and were found eligible, none refused participation, and ultimately 208 were enrolled. Of those enrolled, 99 (48%) were later found to be ineligible due to a negative (n = 87) or missing (n = 12) day 0 slide when slides were re-read for quality control. Therefore, a total of 109 women were included in the study (see Figure 1). Of those included in the study, only 58 (53%) completed the study; 34 (31%) had incomplete follow-up, and 17 (16%) were lost to follow-up after day 0 (see Figure 1). The characteristics of the 92 women (those who completed the study or had incomplete follow-up) who were ultimately included in our analysis are summarized in Table 1. The median age of these women was 20 years [interquartile (IQR) range 18–22 years], with a median gestational age (by last menstrual period) of 22 weeks (IQR range 20–24 weeks). More than half (58%) were primigravid. Most of the women had a very low parasitaemia; the median parasitaemia was 25 parasites/microlitre with an IQR range of 10–56 parasites/microlitre. Use of other malaria control measures such as indoor residual spraying (IRS) and use of an insecticide-treated net (ITN) the night before enrollment, was reported by 45% and 24% of women, respectively. When the characteristics of women included are compared to the 116 women who dropped out or were lost to follow-up, the only significant difference was that women not included were less likely to be primigravid (43%, P = 0.04).

**Table 1:** Characteristics of study participants (N = 92)

**Characteristic**	**n (%)**
Median age (intraquartile [IQR] range)	20 years (18–22 years)
Median gestational age (IQR range)	22 weeks (20–24 weeks)
Median parasitaemia (IQR range)	25 parasites/microlitre (10–56 parasites/microlitre)
Primigravid	47 (58%)
Married	68 (83%)
Educational level (less than 6 years schooling)	23 (66%)
Indoor residual spraying done within last year	36 (45%)
Slept under an insecticide treated net last night	20 (24%)

Of the 58 who completed the study, 15 (26%, 95% confidence interval [CI] [16–38]) had parasitaemic failure during follow-up. Of those with failures, 14 had specimens available for genotyping of the *msp1*, *msp2*, and *glurp* genetic markers. Seven (50%) were recrudescences, while the remaining 7 (50%) were reinfections (Table 2). Adequate parasitic response occurred in 43 (74%, 95% CI [62–84]). The median time to failure was 21 (range 14–35) days. There were no patient or laboratory variables associated with the outcome of failure on bivariate analysis including age, being primigravid, parasitaemia >25 parasites/microlitre (the median), living in a rural area, level of education, use of an ITN the night before, or living in a house sprayed with IRS in the previous year.

**Table 2:** **Results of *dhfr *** **and *dhps *** **genotyping from all day 0 specimens that had complete genotyping available (N = 84)**

**n**	***dhfr *** **mutations**			***dhps *** **mutations**			**SP-IPTp efficacy outcome**		
	**51**	**59**	**108**	**437**	**540**	**581**	APR*	**F†(R‡)**	**U§**
0	–	–	–	–	–	–	N/A	N/A	N/A
1	–	–	+	–	–	–	0	0	1
1	–	+	+	–	–	–	0	0	1
6	–	+	+	+	+	–	2	2 (1)	2
1	+	–	+	–	–	–	1	0	0
1	+	–	+	+	–	–	0	0	1
1	+	–	+	+	+	–	1	0	0
17	+	+	+	–	–	–	11	1	5
2	+	+	+	–	+	–	1	0	1
1	+	+	+	+	–	–	0	1 (1)	0
51	+	+	+	+	+	–	16	9 (4)	26
2	+	+	+	+	+	+	2	0	0

+ = either mutant or mixed genotype.

– = wildtype.

*APR = adequate parasitological response.

†F = parasitological failure.

‡R = reinfection (Only 14/15 patients with failure had specimens available for PCR. One patient with reinfection did not have a complete genotype available).

§U = no outcome available (lost to follow-up or missing day 0 slide).

**Figure 2:**
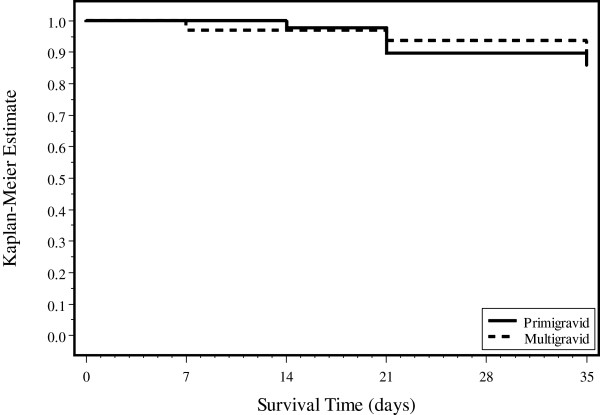
Kaplan Meier survival estimates, stratified by gravidity and PCR corrected (mean time to failure 33.6 days).

Women who completed the study, those with incomplete follow-up, and those with reinfection were included in a survival analysis with the latter two groups censored at the last follow-up visit or the time of reinfection. For these 92 women, the mean time to failure was 33.6 days (standard error 0.6). There was no difference in time to failure in primigravid versus multigravid women (log-rank Chi-sq = 0.20, df = 1, p = 0.65) (see Figure 2). Because of the small sample size and strata, we were unable to adjust for ITN use or IRS.

Of the 84 women who had specimens with complete haplotypes defined as those having haplotypes for *dhfr* loci 51, 59, and 108 and *dhps* loci 437 and 540 (includes specimens from those lost to follow-up after day 0), 20 (24%, 95% CI [16-34]) had the triple *dhfr* mutant only, 7 (8%, 95% CI [4-16]) had the double *dhps* mutant only, and 51 (61%, 95% CI [50–70]) had quintuple mutants. Two (2%, 95% CI [1-8]) of the samples had mutations at codon 581 of *dhps* and both of these samples also had the quintuple mutation, thus for the first time, a sextuple mutant was found in Zambia. There were no mutations identified at codon 164 of *dhfr*.

Of the 58 women with outcomes available, 55 had specimens available for PCR (3 specimens were lost), and 47 of these had full haplotypes available. Among the 47 women who had the outcomes of adequate parasitic response or parasitaemic failure, and complete haplotypes available, the various types *dhfr* and *dhps* mutations and corresponding outcomes are noted in Table 2. Of the 14 specimens with the *dhfr* triple mutant, 2 (14%, 95% CI [2–40%]) had a failure outcome. Of the 5 specimens with the *dhps* double mutant, 2 had the outcome of failure (40%, 95% CI [12–77]). The quintuple mutant was found in 25 specimens, of which 9 (36%, 95% CI [20–55]) were failures. There was no association between quintuple mutation and failure in this small sample. On bivariate analysis the quintuple mutation was not associated with being primigravid, living in a rural area, level of education, use of an ITN the night before, or living in a house sprayed with IRS in the previous year. Surprisingly, the two specimens with the sextuple mutant were from women who had adequate parasitic responses on follow-up.

## Discussion

There was a 26% parasitaemic failure among pregnant, HIV-negative women receiving SP for IPTp in the context of moderate prevalence of the highly resistant quintuple (61%) mutant, and the appearance of the sextuple mutant (2%) among pregnant women in this study in Mansa, Zambia. Despite the moderate prevalence of the highly resistant mutant, IPTp-SP seems to maintain some efficacy in terms of achieving and maintaining parasite clearance for a majority of the women in our study. While this percentage is higher than the 10% treatment failure threshold that WHO recommends for changing first-line anti-malarial policy [26], it is important to note that in the case of IPTp-SP, SP is not being used as a first-line drug to treat malaria, and there are no anti-malarial alternatives to SP for IPTp. Furthermore, among those who failed, there was an average time to failure of 21 days and for those included in survival analysis, a median time to failure of 33.6 days. The current Zambian IPTp-SP policy recommends SP doses spaced at least 4 weeks apart at every antenatal care visit after quickening until delivery [27], suggesting that women could receive a dose at approximately the median time between previous SP and failure. These findings contribute to the current limited literature on how the presence of SP resistance markers in parasites from pregnant women translates into outcomes in terms of recurrent parasitaemia in these same women.

The correlation between prevalence of mutations in *dhfr* and *dhps* among parasites in pregnant women and efficacy of IPTp-SP is not well defined. Most studies of IPTp-SP efficacy have been done in areas with either minimal or very high prevalences of the quintuple mutant [15,25,28,29]. For example in Ghana where triple, but no quintuple mutations have been found, an *in vivo* study confirmed good efficacy of IPTp-SP [30,31]. In Benin where prevalence of the quintuple mutation was less than 10% prior to ITPp-SP treatment, one study found 11% of patients had parasitological failure at 1 month of follow up [32]. The limited sample size of this Benin study prevented reporting on significance of the relationship between the mutated haplotypes and outcomes of low birth weight and maternal anaemia. At the other extreme in terms of frequency of the quintuple mutant is Malawi where 100% penetration of the quintuple haplotype has been observed in parasites from pregnant women [33]. A study in Malawi found that despite the complete penetration of this haplotype, IPTp-SP still had a dose-dependent protective effect among primigravidas for a composite birth outcome of small for gestational age, preterm delivery, or LBW [34]. The present study, with 61% of isolates having quintuple mutation, adds a much-needed data point between these extremes of quintuple mutation prevalence. This study suggests that SP still seems to clear parasitaemia in most pregnant women despite the moderately high prevalence of resistance markers among malaria infected pregnant women. Further supporting the likelihood of continued IPT-SP benefit in the face of resistance, a meta-analysis found that 3 doses versus 2 doses of IPTp-SP resulted in better birth weight outcomes — a finding that was consistent across a range of SP resistance (defined by the presence of molecular markers). The authors postulated that doses beyond the 2^nd^ dose might provide some compensation for a decrease in duration of post-treatment prophylaxis caused by drug resistance [35].

Two studies suggested the possibility that IPTp-SP given in areas with high SP resistance might result in worse outcomes in terms of increased maternal parasite density and placental inflammation [15,36]. They observed that IPTp-SP selected for an increased fraction of parasites with mutations at *dhps* 581 [15]. The present study did find two isolates that harbored both the *dhps* A581G mutation along with the quintuple mutant haplotype; this has never been described in Zambia. Given that Gutman *et al.* have shown some continued benefit for IPTp-SP in the setting of high resistance, the widespread nature of SP resistance, and lack of options for IPTp, further examination of this issue is needed [34].

Of note, this study only looks at parasite clearance in relation to the molecular markers, not birth outcomes such as placental malaria and maternal anaemia. Care must be taken in extrapolating these results to effectiveness of IPTp-SP to prevent malaria-associated adverse birth outcomes such as LBW and neonatal mortality. Also, women not included in the analysis were less likely to be primigravid than those included, and since the benefit of IPTp-SP is most seen among primigravid women, it is possible that observed failure rates may have been higher among those not included.

Furthermore, this study is limited by its small sample size and narrow geographic focus. It is interesting to see that there were no refusals for participation in the study, but there was a high rate of either incomplete or loss to follow up despite monetary incentives to follow-up and reminders from community health workers. It is possible that participants may have agreed to participate as a socially desirable response, but then subsequently dropped out. Furthermore, while reasons for drop-out was not systematically collected, based on the community health worker and study manager notes, a top reason thought to contribute to drop out was agricultural responsibilities elsewhere. It is common for adults to travel seasonally to work their fields, go to the forests to collect caterpillars, or go to fisheries. Additionally, a large contributor to loss in sample size was the high rate of false-positive RDTs. The possibility of false positive RDTs from remaining antigenaemia in those recently treated should be low because women who had taken anti-malarials the month prior to initial presentation were excluded. The RDT and microscopy discrepancy could be due to low-grade parasitaemias observed in the population of asymptomatic pregnant women being studied in Mansa, below the detection limit of microscopy but above the RDT detection level. PCR results of blood from women who had positive RDTs but negative smears would have helped to further explain this issue, but were not available. Quality controls were in place for microscopy, but the study site was remotely located from the reference lab, making on-site confirmation of microscopy reads not possible. So, it is also possible that recurrent parasitaemias, especially if low, were missed.

## Conclusions

This study found a 26% parasitological failure rate for IPTp-SP relative to the moderate 61% prevalence of the quintuple mutant among pregnant women with asymptomatic malaria parasitaemia. The threat of SP resistance looms, and continuous resistance monitoring is needed especially in light of the emergence of the sextuple mutation, but IPTp-SP seems to retain some degree of efficacy in Mansa.

## Abbreviations

ANC: Antenatal care; CI: Confidence interval; dhfr: Dihydrofolate reductase; dhps: Dihydropteroate synthase; IPTp: Intermittent preventive treatment of malaria in pregnancy; IPTp-SP: Intermittent preventive treatment of malaria in pregnancy with sulphadoxine-pyrimethamine; ITN: Insecticide treated nets; IRS: Indoor residual spraying; LBW: Low birth weight; PCR: Polymerase chain reaction; SP: Sulphadoxine-pyrimethamine; WHO: World Health Organization.

## Competing interests

The authors declare no competing interests.

## Authors’ contributions

KT conceived of and designed the study, wrote the protocol, helped with implementation, provided technical expertise, did statistical analysis, and drafted and revised the manuscript. BK was the study coordinator at the study site, implemented the study, and reviewed the manuscript. KM led study implementation, provided technical expertise, and reviewed the manuscript. MN was the key point person for laboratory quality control and laboratory data. ST and SM did the molecular laboratory work, and managed the molecular data, and reviewed the manuscript. RW did statistical analysis and reviewed the manuscript. VC supervised logistics for study implementation and reviewed manuscript. SF provided technical expertise for the design of the study, and reviewed the manuscript. MK provided technical expertise and reviewed the manuscript. AC provided technical expertise, supervised implementation of the study, and reviewed the manuscript. All authors read and approved the final manuscript.
